# Comparison of clinical application of locking plate versus cancellous screw in structural autologous bone grafting during TKA for medial tibial bone defects

**DOI:** 10.3389/fbioe.2026.1685551

**Published:** 2026-01-26

**Authors:** Nanshan Ma, Yiwei Cheng, Haoyuan Ding, Dapeng Han, Sheng Zhong, Jun Xie, Qing Xia, Jing Zhang, Pengfei Xin, Lianbo Xiao

**Affiliations:** 1 Guanghua Hospital Affiliated to Shanghai University of Traditional Chinese Medicine, Shanghai, China; 2 The Research Institute for Joint Diseases, Shanghai Academy of Traditional Chinese Medicine, Shanghai, China; 3 The Affiliated Hospital of Jiangxi University of Chinese Medicine, Nanchang, China

**Keywords:** cancellous screw, locking plate, medial tibial bone defects, structural autologous bone grafting, TKA

## Abstract

**Objective:**

To compare the clinical outcomes of locking plate versus cancellous screw fixation in structural autologous bone grafting for medial tibial bone defects during total knee arthroplasty.

**Methods:**

A retrospective analysis was conducted on 66 patients with medial tibial bone defects who underwent TKA between January 2024 and December 2024. Among them, 34 patients received locking plate fixation (LP group), and 32 patients received cancellous screw fixation (CS group). Postoperative outcomes, including the hip–knee–ankle (HKA), Knee Society Score (KSS) and postoperative complications were recorded and compared to evaluate graft integration and functional recovery.

**Results:**

All patients successfully completed the surgery. At the final follow-up, both groups showed significant improvement in HKA angle, KSS Knee Score, and KSS Function Score compared to preoperative values (P < 0.05). However, no statistically significant differences were observed between the two groups at any time point in terms of KSS Knee and Function Scores (P > 0.05). No postoperative complications occurred in either group during the follow-up period.

**Conclusion:**

Compared with conventional cancellous screw fixation, the use of locking plate combined with structural autologous bone grafting provides similarly favorable clinical outcomes. Furthermore, locking plate offer broader intraoperative applicability and may serve as an effective internal fixation strategy for managing medial tibial bone defects during TKA.

## 1 Introduction

A longstanding controversy persists regarding the optimal reconstruction method for medial tibial bone defects during total knee arthroplasty (TKA) (Kremer et al., 2021). For such bone defects, inappropriate reconstruction strategies can significantly affect the long-term stability of the prosthesis (Ahmed et al., 2008). Common surgical methods include increased tibial resection, bone cement filling, structural bone grafting, and the use of metal augments. Increased tibial resection is only suitable for mild defects (≤2 mm); excessive resection, however, can lead to prosthesis loosening, subsidence, and reduced bone stock (Berend et al., 2010). Bone cement filling is not limited by defect morphology and is technically simple, but it is prone to fracture due to the stiffness mismatch between the cement and host bone, and is not conducive to long-term bone preservation (Huten et al., 2021). Metal augments are costly and require intraoperative reshaping of the defect to match the block, resulting in further bone loss (Brand et al., 1989). In contrast, autologous structural bone grafting remains a preferred method for tibial plateau bone defect reconstruction due to its ease of harvest, good biocompatibility, and high bone union rate (Dorr et al., 2006). In structural bone grafting, compared with allograft bone, autologous bone grafting can avoid delayed bone union and bone resorption collapse, and also circumvent the risks of infection and disease transmission (Dennis, 2002). Once fused, it can become part of the medial tibia, allowing load transmission in a manner closer to the physiological state (Iwase et al., 2022).

Autologous bone grafting is increasingly utilised owing to its favourable biological properties and implant-compatible mechanical characteristics (Adam and Damian, 2006). However, there remains no clinical consensus regarding the optimal fixation methods for structural autologous grafts in this setting. Previously, we proposed an innovative reconstruction strategy specifically for patients with medial tibial bone defects: during TKA, this approach utilises a locking plate combined with structural autologous bone graft to repair tibial plateau bone loss. Finite element analysis has confirmed that this technique effectively maintains postoperative knee stability under both weight-bearing and rotational load*s* (Ma et al., 2025). Building upon that foundation, we conducted a retrospective clinical study comparing the clinical outcomes of the locking plate technique with the conventional cancellous screw fixation method for structural autologous bone grafting in medial tibial bone defects. We assessed differences in postoperative pain relief, functional recovery, and other clinical outcomes between the two surgical strategies. Our aim was to provide a clinically evidence-based novel reconstruction option for medial tibial bone defects.

## 2 Methods

### 2.1 Patient data

A retrospective study was conducted on 66 patients with bone defect who underwent TKA surgery from January 2024 to December 2024. Thirty-four patients underwent bone defect reconstruction using locking plate during TKA (LP Group), while 32 patients received fixation with cancellous screws during TKA (CS Group). All surgeries were performed by the same lead surgeon and all included patients were of Han ethnicity. The study was approved by the Ethics Committee of Guanghua Hospital Affiliated to Shanghai University of Traditional Chinese Medicine (Approval No. 2025-K-78).

### 2.2 Inclusion and exclusion criteria

#### 2.2.1 Inclusion criteria

Patients with end-stage knee osteoarthritis (KOA), classified as Kellgren–Lawrence grade IV, and undergoing primary total knee arthroplasty.

Radiographic evidence indicates a medial tibial bone defect classified as Rand type II/III, which is a non-contained bone defect.

Persistent knee pain without improvement following ≥6 months of conservative treatment.

#### 2.2.2 Exclusion criteria

Presence of neuromuscular or musculoskeletal disorders or active knee joint infection before surgery.

Patients who develop postoperative complications necessitating a second surgery.

Poor follow-up adherence or incomplete follow-up data.

### 2.3 Surgical technique

Both groups of patients underwent primary TKA. Intraoperatively, tibial stem extender was used in all cases to maintain the initial stability of the knee joint after surgery and assist patients in early weight-bearing rehabilitation training. Structural autogenous bone grafts were fashioned using resected tibial and femoral bone fragments, tailored to match the specific morphological deficits of the tibial plateau. The sclerotic bone on the medial tibial plateau was meticulously debrided to create a viable freshened graft bed for implantation.

#### 2.3.1 LP group

**FIGURE 1:**
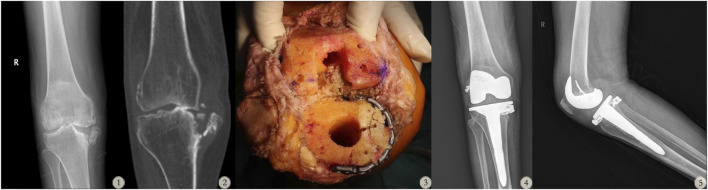
A 76-year-old female with Rand III type bone defect in the right knee. (1–2: Preoperative X-ray and CT scan of the patient’s right knee. 3: Intraoperative images showing the use of a tibial stem extender combined with a locking screw-plate to address the bone defect during TKA surgery. 4–5: Postoperative follow-up X-rays of the patient’s right knee.)

After placement of the autologous bone graft on the proximal medial tibial plateau, a locking plate was applied to achieve stable fixation. The graft autologous bone height was precisely adjusted to match the lateral tibial plateau, thereby restoring balanced flexion-extension gaps and ensuring the restoration of the normal joint line, shown in Figure 1.

#### 2.3.2 CS group

**FIGURE 2:**
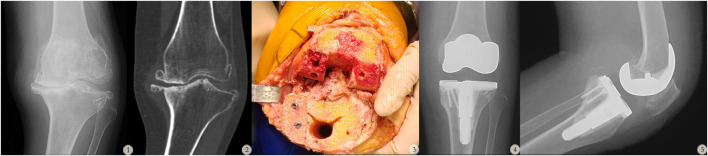
A 59-year-old female with Rand II type bone defect in the left knee. (1–2: Preoperative X-ray and CT scan of the patient’s left knee. 3: Intraoperative images showing the use of a tibial stem extender combined with two cancellous screws to address the bone defect during TKA surgery. 4–5: Postoperative follow-up X-rays of the patient’s left knee.)

One or more cancellous screws were used to fix the graft autologous bone to the medial tibial defect, with the number of screws determined by intraoperative stability requirements. Other intraoperative procedures were identical to those in the LP group shown in Figure 2.

Tourniquet was routinely applied preoperatively to achieve hemostasis, aiming to reduce intraoperative bleeding and minimize infection risk. In addition, all patients received a prophylactic intravenous infusion of 1 g cefazolin sodium on the day of surgery to prevent infection. Postoperative follow-up was performed regularly, and timely interventions were implemented in cases of nonunion. The PS prostheses used were all Persona® (Zimmer Biomet, Indiana, United States).

### 2.4 Observational items and methods

#### 2.4.1 Radiologic outcomes assessment

All patients underwent preoperative and postoperative knee radiographs. The primary imaging parameter recorded at baseline and at the 6-month follow-up was the hip–knee–ankle angle (HKA), which was measured in the standing (weight-bearing) position to assess the mechanical alignment correction achieved after TKA.

#### 2.4.2 Clinical outcomes assessment

Clinical outcomes were evaluated using the Knee Society Score (KSS) (Insall et al., 1989), recorded preoperatively and at 1, 3, and 6 months postoperatively. This early postoperative assessment focused on pain intensity and functional recovery.

The KSS consists of two distinct components:

KSS Knee Score: Includes evaluation of postoperative pain, alignment, stability, and range of motion (ROM). Notably, all deduction items are consolidated under the stability domain in this study.

KSS Function Score: Assesses walking ability and stair-climbing capacity, reflecting the level of functional recovery following TKA.

### 2.5 Statistical analysis

The statistical analysis was conducted using SPSS 26 statistical software (IBM Corporation, United States). The Shapiro-Wilk test was applied to determine if the variables were normally distributed. Variables that were normally distributed are reported as the Mean ± SD, while those that were not normally distributed are reported as the median with interquartile range. For comparing normally distributed continuous variables between groups, independent samples t-tests were used. Conversely, the Mann-Whitney U test was applied for continuous variables that were not normally distributed. Categorical variables were compared using the chi-square test. A statistically significant result was defined as P < 0.05 in this study.

## 3 Result

### 3.1 General result

A total of 66 eligible patients were enrolled in this study—34 in the LP group and 32 in the CS group. In the LP group, there were 13 males and 21 females, with a mean age of 69.21 ± 4.9 years. The HKA averaged 9.13 ± 5.47°, including 19 cases of Rand II type and 15 cases of Rand III type bone defects. In the CS group, there were 13 males and 19 females, with a mean age of 68.66 ± 5.96 years. Their preoperative HKA angle averaged 9.09 ± 5.91°, including 22 cases of Rand II type and 10 cases of Rand III type bone defects. The operative time was significantly shorter in the CS group compared to the LP group (P < 0.05); all other baseline characteristics showed no statistically significant differences between groups (P > 0.05). Detailed demographics are summarized in Table 1.

**TABLE 1:** Characteristics of included patients.

Characteristics	LP group (n = 34)	CS group (n = 32)
Age	69.21 ± 4.9	68.66 ± 5.96
Gender		
Male	13 (38.2%)	13 (40.6%)
Female	21 (61.8%)	19 (59.4%)
BMI	25.1 ± 2.49	25.68 ± 2.37
HKA (preoperative)	9.13 ± 5.47°	9.09 ± 5.91°
Rand classification		
Rand II	19	22
Rand III	15	10
Surgical time(min)	105.59 ± 7.45	89.38 ± 11.34*
Hospital day	8.38 ± 1.99	8.56 ± 1.63

*P < 0.05.

### 3.2 Outcome measures

Postoperative follow-up showed that both groups experienced significant improvement from baseline in all domains of the KSS Knee Score at 1, 3, and 6 months post-surgery—including pain, joint stability, and range of motion. There was a statistically significant difference between the last follow-up and the preoperative period (P < 0.05). However, no statistically significant differences were detected between the LP and CS groups in the magnitude of improvement in these three domains (P > 0.05), shown in Table 2.

**TABLE 2:** Change in KSS knee score.

Time point	LP group			CS group		
	Pain	Stability	ROM	Pain	Stability	ROM
Pre-OP	10 (0, 20)	0.65 ± 13.43	14 (12, 15)	10 (0, 20)	−0.78 ± 11.58	14 (12.25, 15.75)
1M Post-OP	20 (20, 30)	14 (10, 18)	17 (16, 17)	25 (20, 30)	13.5 (9, 17)	17 (16, 17.75)
3M Post-OP	30 (20, 30)	13.85 ± 4.64	18 (18, 19)	30 (22.5, 30)	13.59 ± 4.61	19 (18, 19)
6M Post-OP	30 (20, 36.25)	15.29 ± 4.33	18.5 (18, 20)	30 (30, 35)	14.97 ± 4.36	19.5 (18.25, 20)

**FIGURE 3:**
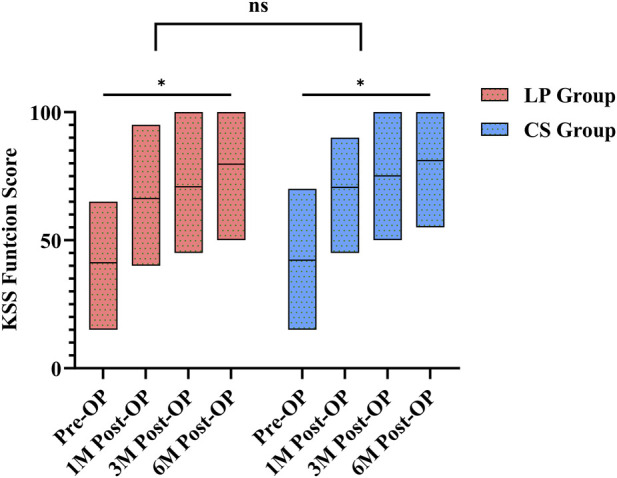
Change in KSS function score.

Similarly, KSS Function Score improved significantly from preoperative values in both groups at all postoperative time points, indicating that both fixation methods effectively promote functional recovery, and the difference between the last follow-up and the preoperative period was statistically significant (P < 0.05). Intergroup comparison revealed no significant difference in the degree of improvement between LP and CS groups (P > 0.05), shown in Figure 3.

**FIGURE 4:**
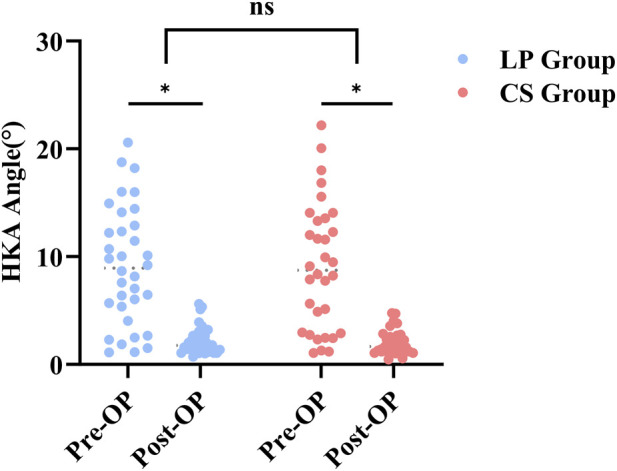
Change in HKA angle.

Radiographic analysis demonstrated a significant reduction in postoperative HKA angle compared to preoperative values in both groups (P < 0.05), indicating effective restoration of lower limb alignment with both structural grafting and fixation techniques. The proportion of patients achieving postoperative HKA within 3° was 79.4% in the LP group and 84.4% in the CS group, reflecting comparable rates of alignment correction, with no statistically significant difference between the groups, shown in Figure 4.

At the 6-month postoperative follow-up, bone healing was observed in the grafted regions of both groups. No signs of prosthetic loosening or displacement were detected, and there was no evidence of bone resorption or nonunion at the graft sites. Additionally, no indications of prosthetic subsidence, infection, or related complications were observed.

## 4 Discussion

Reconstruction strategies vary according to the severity of the bone defect. Mild defects can be managed by increased bone resection or bone cement filling techniques. However, for severe tibial bone defects (>10 mm), resection down to the defect level is not recommended, as it may compromise bone support, reduce the contact area, and elevate the mechanical load on the residual bone (Chon et al., 2021; Harada et al., 1988). Traditionally, autologous bone grafting refers to a technique in which bone is harvested from non-essential donor sites (e.g., the iliac crest) and transplanted into the same individual to fill bone defects and induce osteogenesis, which is typically indicated for small defects (Van et al., 2000). In recent years, however, several studies have confirmed that this approach can also be successfully applied to large bone defects, providing both structural support and biological integration. Dewidar et al. demonstrated that autologous structural bone grafting is effective for uncontained tibial defects larger than 10 mm. Intraoperative harvest of local autografts from the resected bone during TKA avoids donor-site morbidity in other anatomical regions (Dewidar et al., 2024). Owing to its strong osteogenic potential, excellent biocompatibility, reliable load-bearing capacity, bone stock preservation, and cost-effectiveness, autologous structural bone grafting has been extensively adopted in clinical practice (Lei et al., 2019).

Clinically, autologous bone grafts are often fixed with cancellous screws, which provide rigid initial fixation and facilitate the maintenance of long-term alignment (Bauman et al., 2009). However, screw fixation alone may result in irregular bone surfaces, unsatisfactory reduction angles, and graft rotation; the use of multiple screws may even cause graft fracture (Yang et al., 2024; Watanabe et al., 2001). Locking plates are widely used for internal fixation in tibial fractures and high tibial osteotomy (HTO) (Blažević et al., 2022; Chen et al., 2018; Berthe et al., 2025). The combination of locking plate and autologous structural bone grafting during TKA can provide robust lateral support, resist shear stress, restore the tibial plateau slope and height, correct varus deformity, and preserve more bone stock, thereby optimizing residual bone reserves. Biomechanical analyses have shown that this technique confers superior mechanical performance in resisting bending and shear stresses, achieves more uniform stress distribution, and alleviates stress concentration at the screw-bone interface (Mitrogiannis et al., 2024; Yan et al., 2024). A retrospective analysis by Wang et al. (2022) also verified the efficacy of this technique for primary TKA in patients with severe medial tibial bone defects. In their study of 21 patients with Rand type IIIb–IVb defects, all grafts achieved bony union, with significant postoperative improvement in knee function; no cases of prosthesis loosening or infection were reported.

In this study, autologous bone grafts harvested from femoral or tibial plateau osteotomy were contoured to fill the defects in both groups. The CS group underwent cancellous screw fixation, while the LP group received locking plate fixation combined with screws. Follow-up revealed no significant differences in KSS scores between the two groups, with both achieving favorable functional recovery and joint stability. No prosthesis loosening, infection, or periprosthetic fractures were observed in the short term, and obvious bone healing was noted at the graft sites. Intraoperatively, we found that compared with screw fixation alone, locking plates had broader applicability in autologous structural bone grafting for tibial bone defects. First, plates can be flexibly contoured according to the defect size and morphology to reconstruct the tibial margin. Second, locking plates have lower requirements for the integrity and size of autologous bone grafts. When cancellous screw fixation is used alone for severe defects, it is often difficult to obtain intact autologous grafts of matching size, and fragmented or small bone grafts cannot maintain screw fixation stability (Lin et al., 2022). In contrast, autologous bone grafts from the resected femur or tibia can be directly used for grafting with locking plate fixation, which is particularly advantageous for severe bone defects, especially Rand type III defects.

This technique also has certain limitations: it requires a longer operative time, more extensive soft tissue dissection, and may be associated with a higher infection risk, as well as medial prosthesis loosening. Intraoperatively, an appropriately sized plate should be selected and properly contoured; curved contouring helps preserve more bone tissue, restore the original joint structure, and optimize load transfer. Plates that are too thin may lack sufficient medial support, while excessively thick plate may impede soft tissue closure. Screw placement should avoid contact with the prosthesis or stem extender, a complication that is less likely to occur with cancellous screw fixation alone. Additionally, finite element analysis indicates that the stiffness mismatch between the locking plate and autologous bone graft may induce stress shielding, which reduces mechanical stimulation to the graft and potentially increases the risk of delayed union (Ma et al., 2025).

In summary, locking plate fixation for autologous structural bone grafting can provide stable fixation in the reconstruction of complex or large-volume tibial bone defects. It has low requirements for the integrity of autologous bone grafts and offers excellent intraoperative adaptability, rendering it a viable and effective option for the reconstruction of medial tibial bone defects during TKA.

## 5 Conclusion

Compared with cancellous screw fixation combined with structural autologous bone grafting, locking plate fixation yields similarly favorable outcomes in postoperative pain relief and functional recovery. Moreover, its broader intraoperative adaptability indicates that this technique may represent a valuable alternative for the management of tibial bone defects.

## 6 Limitation

This study is a single-center retrospective analysis with a relatively small sample size, which may limit the statistical power and generalizability of the findings. Moreover, the follow-up period was confined to short- and mid-term outcomes, without long-term validation. In particular, whether the stiffness mismatch between the locking plate and the autologous bone may lead to stress shielding and hinder bone integration remains uncertain. Therefore, future studies with larger sample sizes, multi-center participation, and longer follow-up are warranted to strengthen the reliability and applicability of these findings.

## Data Availability

The raw data supporting the conclusions of this article will be made available by the authors, without undue reservation.
